# Full Spectrum of Reported Symptoms of Bilateral Vestibulopathy Needs Further Investigation—A Systematic Review

**DOI:** 10.3389/fneur.2018.00352

**Published:** 2018-06-04

**Authors:** Florence Lucieer, Stijn Duijn, Vincent Van Rompaey, Angelica Pérez Fornos, Nils Guinand, Jean Philippe Guyot, Herman Kingma, Raymond van de Berg

**Affiliations:** ^1^Division of Balance Disorders, Department of Otorhinolaryngology and Head and Neck Surgery, Maastricht University Medical Center, School for Mental Health and Neuroscience, Maastricht, Netherlands; ^2^Faculty of Health, Medicine and Life Sciences, University of Maastricht, Maastricht, Netherlands; ^3^Department of Otorhinolaryngology and Head and Neck Surgery, Antwerp University Hospital, Faculty of Medicine and Health Sciences, University of Antwerp, Antwerp, Belgium; ^4^Service of Otorhinolaryngology Head and Neck Surgery, Department of Clinical Neurosciences, Geneva University Hospitals, Geneva, Switzerland; ^5^Faculty of Physics, Tomsk State Research University, Tomsk, Russia

**Keywords:** bilateral vestibulopathy, symptoms, dizziness, vertigo, imbalance, oscillopsia, bilateral vestibular hypofunction, patient-reported outcome measures

## Abstract

**Objective:**

To systematically review the symptoms reported by patients with bilateral vestibulopathy (BV) in clinical studies and case reports. This would serve as the first step in establishing a validated patient-reported outcome measures (PROM) for BV.

**Methods:**

A search on symptoms reported by patients with BV was performed in PubMed, and all publications covering these symptoms were included. Exclusion criteria comprised reviews and insufficient details about the frequency of occurrence of symptoms.

**Results:**

1,442 articles were retrieved. 88 studies were included (41 clinical studies, 47 case reports). In consensus, 68 descriptions of symptoms were classified into 6 common and generic symptoms. Frequency of symptoms in clinical studies and case reports were reviewed, respectively; imbalance (91 and 86%), chronic dizziness (58 and 62%), oscillopsia (50 and 70%), and recurrent vertigo (33 and 67%). BV could be accompanied by hearing loss (33 and 43%) and tinnitus (15 and 36%). 15 clinical studies and 10 case reports reported symptoms beyond vestibular and hearing deficits such as limited social activities, depression, concentration, and memory impairment and reduced quality of life in general.

**Conclusion:**

The literature on BV symptomatology mainly focuses on classic symptoms such as imbalance and oscillopsia, while only few report additional symptoms such as cognitive memory impairment and performing dual tasks. In fact, none of the reviewed clinical studies and case reports provided a comprehensive overview of BV symptoms. To develop a validated PROM, qualitative research using semi-structured and unstructured interviews is needed to explore the full spectrum of BV symptoms.

## Introduction

Bilateral vestibulopathy (BV) is a heterogeneous chronic condition in which the vestibular function is bilaterally absent or reduced (1). BV can be due to a dysfunction of the vestibular organs, nerves, and/or the brain (1, 2). In 2017, consensus was reached by the Classification Committee of the Bárány Society about the diagnostic criteria of BV (3). Symptoms for diagnosis include unsteadiness when walking or standing, movement-induced blurred vision/oscillopsia during walking or quick head movements, or worsening of unsteadiness on uneven ground and/or darkness (1, 3, 4). However, clinical experience and current literature point to a wider variety of symptoms (5). For example, many patients report a negative impact on physical and social functioning, and compromised cognitive abilities (6–8).

At this moment, questionnaires like the dizziness handicap inventory (DHI) exist to quantify the dizziness symptoms. However, these questionnaires are not specific for vestibular loss (9). Therefore, the objective of this study was to systematically evaluate the nature and frequency of BV symptoms reported in clinical studies and case reports. This would serve as the first step in establishing validated patient-reported outcome measures (PROM) specifically for patients with BV (10). This is a tool to measure patient perceptions of their own functional status and well-being (11).

## Methods

### Information Source and Search Strategy

A systematic literature search was performed according to the PRISMA statement in the bibliographical database PubMed based on the following keywords: Bilateral[All Fields] AND ((Vestibular[All Fields] AND (Hypofunction[All Fields] OR Failure[All Fields] OR Loss[All Fields])) OR Vestibulopathy[All Fields]) (12). At the beginning, a full search was performed followed by entering some additional restrictions. The outcomes of this selection were first screened by title and then also by abstract. The last selection included a complete reading of the articles found. The selections and full article analysis was performed by the first two authors (Florence Lucieer and Stijn Duijn).

### Selection Criteria and Study Selection

A requirement in the basic search was that all articles were written in English. All publications reporting on symptoms of patients with BV were considered, including all types of BV (central, peripheral, or mixed etiology).

#### Title Screening

Articles were excluded in case of animal studies, if they did not report on BV, if they did not report on symptoms of patients with BV, if no or insufficient details on the frequency of symptoms were available, and in case of systematic reviews, comments, errata, or books.

#### Abstract Screening

Exclusion criteria were the same as for title screening. In case an abstract was not available, the full article was screened to search for any of the exclusion criteria.

#### Full-Text Review

In case the full article was not available, the article was excluded. Whenever the inclusion or exclusion criterion of an article included only “unsteadiness” or “oscillopsia,” or “unsteadiness and oscillopsia,” the article was excluded, so few reported symptoms could lead to a bias of interpretation of the frequency of the symptoms.

### Data Collection Process and Risk of Bias Assessment

Title selection, abstract selection, full read through selection, and the analysis of the included articles were performed by the first and second author (Florence Lucieer and Stijn Duijn). The list of articles found in the PubMed search was exported to EndNote X8 for Windows and Mac (Clarivate Analytics, Philadelphia). This Endnote library was then used to perform the title, abstract, and full article selection by the two researchers separately. After the title and abstract screening the articles of both researchers were combined and duplicates were removed. After full article selection, articles were combined. Discrepancies between the two authors were discussed and a consensus was reached about selecting the articles for data analysis. A Cohen’s kappa of the full article selection was calculated to evaluate the interrater reliability (13).

The nature and frequency of symptoms and the total number of patients were extracted by the first two authors and compiled using Microsoft Excel. If only percentages were reported, the authors were contacted to get the exact number of patients. The method of symptom collection in the articles was collected to assess risk of bias of the studies. Clinical studies and case reports were analyzed separately.

### Qualitative Data Synthesis

The primary outcome measure of this systematic review was a detailed overview regarding nature and frequency of symptoms of BV as reported in clinical studies and case reports. Three authors (Florence Lucieer, Stijn Duijn, and Raymond van de Berg) reviewed the list of descriptions. In consensus, these descriptions were categorized into common symptoms. The frequency of these common symptoms was calculated.

## Results

### Search Process

**Figure 1:**
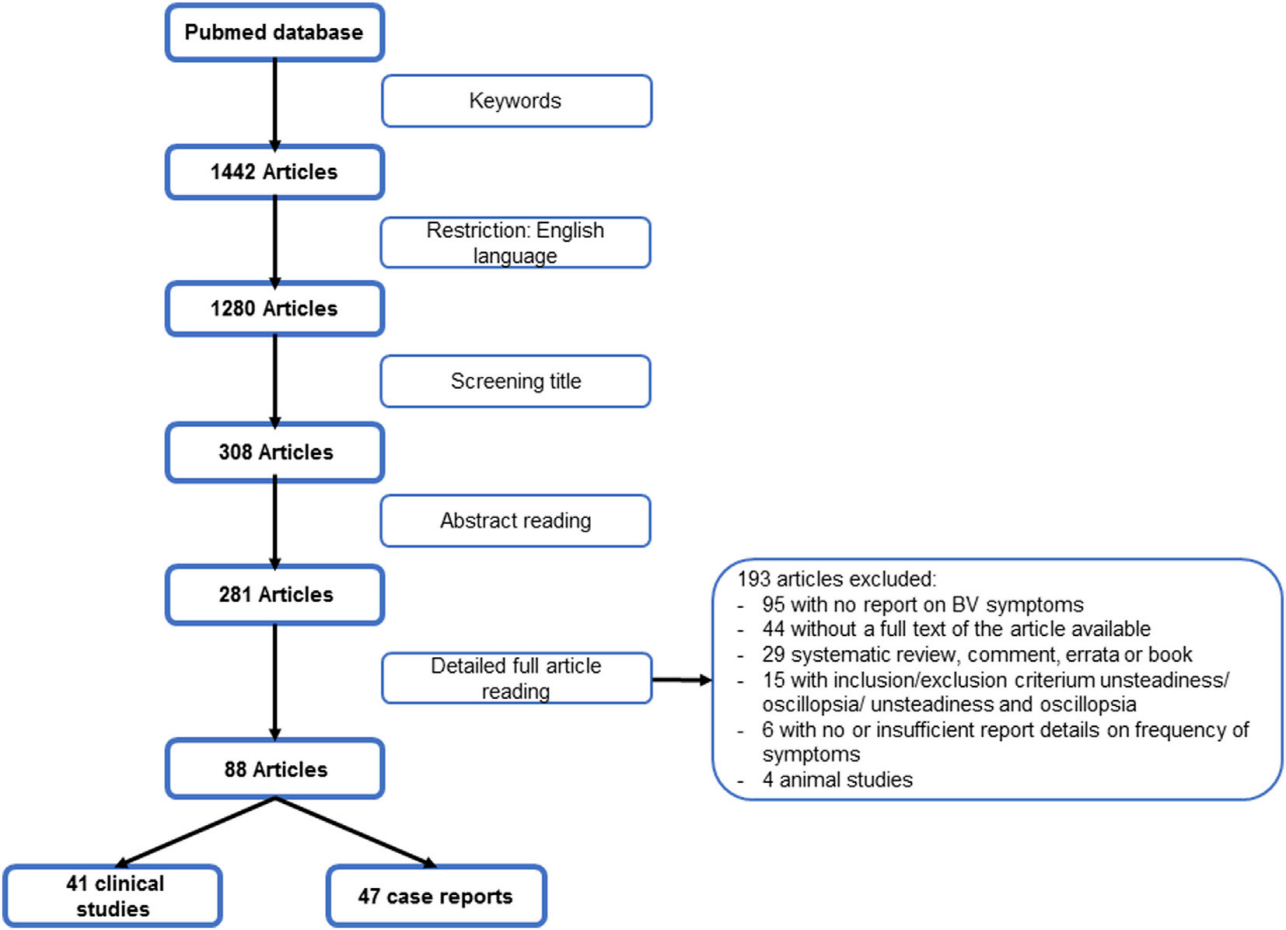
Flowchart search strategy.

The search was conducted in the bibliographical database PubMed on October 25, 2017. The initial search resulted in 1,442 publications. For a schematic overview of the search strategy, see Figure 1. A high level of interrater reliability was reached on the full article selection with a Cohen’s kappa of 0.98. Finally, 1,385 unique patients from 41 clinical studies and 86 patients from 47 case reports were included (2, 5, 14–99). The characteristics of these articles can be found in Tables A1 and A2 in Supplementary Material.

### Demographic Data of the Articles

Twenty-two of the clinical studies were European, 12 North-American, 4 Asian, 3 Australian, and 1 South-American, dated between 1984 and 2017. Twenty-one case reports were European, 13 North-American, 11 Asian, 1 Australian, and 1 South-American, originated from 1985 until 2017.

### Classification of Symptoms

Sixty-eight descriptions of BV symptoms were retrieved. In consensus, these descriptions were classified into six universal symptoms: imbalance (including worsening in darkness or on uneven grounds), chronic dizziness, oscillopsia, recurrent vertigo, hearing loss, and tinnitus. Data from these six universal symptom classes can be found in Table A3 in Supplementary Material.

#### Clinical Studies

Symptoms found in clinical studies are presented in Table 1. Two times, two publications were merged in the results because of reporting duplicate data (24, 74, 97, 98). Imbalance was the most frequent symptom (91.4%). Fifteen clinical studies reported additional symptoms, for example, socio-economic impacts, depression, cognitive impairment, and increased risk of falling (5, 16, 27, 32, 43, 45, 54, 60, 63, 75, 76, 81–83, 90).

**Table 1:** Nature and frequency of most common symptoms reported by patients with bilateral vestibulopathy in clinical studies.

Symptom	n/t	Reported (%)
Imbalance Worse in darkness On uneven ground	1,025/1,121 110/115 12/12	91.4% 95.7% 100%
Chronic dizziness	86/149	57.7%
Oscillopsia	559/1,116	50.1%
Recurrent vertigo	267/808	33.0%
Hearing loss	256/787	32.5%
Tinnitus	59/398	14.8%

*n, number of patients reporting the symptom; t, total number of patients in subset*.

#### Case Reports

Symptoms found in case studies are presented in Table 2. Imbalance was the major symptom (86.1%). Ten case reports reported additional symptoms like sleep disturbances, unable to perform daily chores, and appearing to be depressed (18, 28, 30, 62, 67, 68, 70, 89, 91, 94). For a complete overview of additional symptoms in clinical studies and case reports, see Table 3.

**Table 2:** Nature and frequency of most common symptoms reported by patients with bilateral vestibulopathy in case reports.

Symptom	n/t	Reported (%)
Imbalance Worse in darkness On uneven ground	62/72 28/42 7/13	86.1% 66.7% 53.8%
Chronic dizziness	21/34	61.8%
Oscillopsia	40/57	70.2%
Recurrent vertigo	36/54	66.7%
Hearing loss	30/70	42.9%
Tinnitus	10/28	35.7%

*n, number of patients reporting the symptom; t, total number of patients in subset*.

**Table 3:** Additional symptoms in bilateral vestibulopathy reported in articles.

	Additional symptoms
Clinical studies	– Prevented from doing things they could otherwise do (90) – Change or limited social activities (90) – Missed days from work or school (90) – Depression (90) – Tiredness (5) – Concentration difficulties (5) – Memory impairment (5) – Disorientation in space (5) – Muscular pain (5) – Ashamed (5) – Needing walking aid (16, 27) – Falling (5, 19, 27, 43, 54, 75, 76, 81–83) – Sleep efficiency was reduced (63) – Headache (32)

Case reports	– Unable to work (30) – Headaches (62, 68, 70) – Occasional sleep disturbances (62) – Difficulties with functional tasks in daily living (62) – Needing walking aid (68) – Anosmia (18) – Ageusia (18) – Dysarthria (18) – Tired (89, 91) – Unable to perform daily chores (28) – Appeared depressed (28, 67, 91) – Hitting walls (28) – Falling (28, 91) – Fear of falling (94) – Avoided crowded places and the use of public transport (94) – Hypersensitive to changes in environment (94) – Dizzy when exposed to lights other than natural white light (94) – Dizziness worsened in a crowded place such as a shopping mall or after working several hours on a computer (94) – Difficulty driving over rough surfaces (91) – More anxious (91) – Feelings of disorientation (91)

## Discussion

After a structural review until October 2017, 41 clinical studies and 47 case reports were reviewed to determine the nature and frequency of symptoms experienced by patients with BV. No study was identified that created an inventory of patient-reported outcomes. Almost all patients suffered from imbalance, chronic dizziness, and oscillopsia, next to other inner ear problems like hearing loss and tinnitus. The high rate of imbalance and oscillopsia comply with the recent diagnostic criteria for BV (3). However, additional symptoms were rarely mentioned.

Hearing loss and tinnitus most likely shared the same etiology as BV or resulted from aging, rather than from vestibular dysfunction. A typical example for this is aminoglycoside toxicity is a well-known cause of sensorineural hearing loss and vestibular dysfunction (2, 97, 100). In addition, the average patient with BV in the literature was 60–62 years old and the prevalence of hearing loss in the United States is 25.1 and 42.7% for the age groups 55–64 and 65–84, respectively (101). The same conclusion could be drawn for recurrent vertigo. Vertigo is most likely an expression of the shared etiology, but is not the result of BV since an absent or reduced vestibular function is most likely not the cause of attacks of vertigo (2, 4).

Only 15 clinical studies and 10 case reports reported additional symptoms. The existence of these symptoms is supported by other literature that suggested that patients with BV could also suffer from cognitive deficits (7, 8), autonomic (102–104) and psychological symptoms (6, 105, 106), tiredness (6), visually induced dizziness (7, 107, 108), and impaired quality of life (6, 90, 106). Unfortunately, these symptoms could not reliably be quantified since they were not often mentioned in clinical studies and case reports. At this moment, it is uncertain why these symptoms were reported so infrequently. It could be hypothesized that these symptoms did have a low occurrence, that they were not part of routine history taking, or that patients were not aware of the link between their vestibular deficit and these types of symptoms (4). Therefore, structured patient interviews with open-ended questions should be conducted in which patients with BV are specifically asked to describe all of their symptoms and thereby evaluate in which words they describe their own symptoms, to determine the nature and frequency of all symptoms related to BV. This is necessary to develop PROM for BV (10).

At this moment, vestibular specific PROM exist like the DHI (109) and the vestibular disorders activities of daily living scale (VADL) (110). The DHI evaluates different aspects of vestibular complaints (function, physical, and emotional) and the VADL assesses the independency in activities of daily living. However, both questionnaires only focus on balance and do not assess the classic BV symptoms like oscillopsia, recurrent vertigo, hearing loss, and tinnitus. Therefore, there is also a need for PROM for BV specifically.

Several limitations of this systematic review were identified. Almost all articles gathered data differently and many of them were of retrospective nature. It was uncertain whether all publications used an open interview and whether all symptoms were explicitly mentioned or not. In addition, it was not possible to determine whether symptoms were mentioned but denied, or not even mentioned at all. Moreover, patients can describe the same complaint (e.g., dizziness and vertigo) differently. As a result, the same complaints could be categorized into different universal symptoms (4, 111). In addition, different articles used various diagnostic criteria for BV, resulting in a heterogeneous patient population, which made direct comparison of the patient population between articles difficult. Furthermore, it is known that BV is often misdiagnosed and missed (4, 90). This implies that even the percentages mentioned in literature do not reflect the real prevalence of the symptoms. Finally, only three clinical studies and one case report measured quality of life (e.g., DHI and HADS), therefore, outcomes could not be pooled for analysis (27, 30, 41, 42).

In this review, clinical studies and case reports provided complimentary information. Clinical studies were better in quantifying the established symptoms and case reports were better in giving an overview of the array of symptoms.

In the future, a qualitative research model would be of added value. This would allow getting a clear overview of all symptoms patients with BV experience, including additional symptoms.

## Conclusion

Current literature on BV symptomatology mainly focuses on classic symptoms such as imbalance and oscillopsia, while only a few report additional symptoms such as cognitive memory impairment and dual tasking. In fact, none of the reviewed clinical studies and case reports provided a comprehensive overview of BV symptoms. To develop validated PROM, a qualitative research using semi-structured and unstructured interviews is needed to explore the full spectrum of BV symptoms.

## Author Contributions

All authors contributed extensively to the work presented in this paper. FL and SD conducted the analysis and wrote the manuscript. RB supervised the writing and edited the manuscript. HK supervised the writing and reviewed the manuscript. VR, AF, NG, and JP reviewed the manuscript.

## Conflict of Interest Statement

The first author was supported through funding of MedEl. The remaining coauthors declare that the research was conducted in the absence of any commercial or financial relationships that could be construed as a potential conflict of interest. The reviewer DS and handling Editor declared their shared affiliation.
