# Supplementary material for: Full Spectrum of Reported Symptoms of Bilateral Vestibulopathy Needs Further Investigation—A Systematic Review
Source: Front Neurol. 2018 Jun 4;9:352. doi: 10.3389/fneur.2018.00352 (PMC5994412; doi:10.3389/fneur.2018.00352)
Supplement: Supplementary file 1 [file Data_Sheet_1.PDF]

SUPPLEMENTARY MATERIAL

Table A1 | Specification of the included clinical studies

Reference	No. patients with BV	Continent study	Research topic	Etiologies of BV	Method of symptom collection
Swanenburg et al. (82)	18	European	Gait and falls	Idiopathic, ototoxic medication, meningitis, genetic confirmed spinocerebellar atrophy, autoimmune inner ear disease,	Not specified
Schniepp et al. (76)	55	European	Gait and falls	Idiopathic, ototoxic medication, Menièrés disease	Standardized interview
Moon et al. (66)	15	Asian	Video head impulse test	Idiopathic, sudden sensorineural hearing loss, bilateral chronic otitis media	Retrospective review of clinical records
Deroualle et al. (35)	23	European	Visuo-spatial perspective taking and embodiment	Idiopathic	Patient reported
de Waele et al. (34)	8	Australian	Suppression head impulse paradigm	Not specified	Patient reported
Schlick et al. (75)	21	European	Fall risk	Not specified	Questionnaire
Martin et al. (63)	9	European	Human circadian rhythmicity in BV	Not specified	Questionnaire and patient reported
Lucieer et al. (2)	154	European	Etiologies, clinical subtypes of BV and diagnostic process	Idiopathic, genetic disorders, Menièrés disease, ototoxic medication, infectious diseases, neurodegenerative diseases	Retrospective review of clinical records
Choi et al. (32)	18	Asian	Diagnosing combined peripheral and central BV	Wernicke encephalopathy, infarction, cerebellar ataxia and BV (CABV), cerebellar ataxia with neuropathy and bilateral vestibular areflexia syndrome (CANVAS), cerebellopontine angle tumor, cerebral superficial siderosis, vestibular schwannoma	Not specified
Miffon and Guyot (5)	19	European	Symptoms of patients with BV	Idiopathic, ototoxic medication, neurofibromatosis	Semi-structured interview
Jandl et al. (52)	23	European	Cerebellar activity during spatial navigation and visual memory	Idiopathic, ototoxic medication, vestibular neuritis, Menièrés disease	Patient reported
Jansen et al. (53)	12	European	Dopamine-receptors in BV	Idiopathic, Menièrés disease	Patients were asked for presence of oscillopsia
Ward et al. (90)	12	North-American	Prevalence of functional impact of BV	Idiopathic, ototoxic medication, Menièrés disease, head and neck trauma	Interview
Suarez et al. (81)	8	South-American	Behavior of postural response in elderly with BV	Not specified	Not specified
Kapoula et al. (58)	11	European	Postural control	Idiopathic	Not specified
Ghulyan-Bedikian et al. (41)	28	European	Head-position based, tongue-placed electrotactile feedback	Not specified	Patient reported
Fujimoto et al.(40)	43	Asian	Clinical features of BV affecting superior vestibular nerve and inferior vestibular nerve	Idiopathic, Menièrés disease, vestibular neuritis, migraine-associated vertigo, acoustic neuroma, delayed endolymphatic hydrops, benign paroxysmal positional vertigo, peripheral sensory polyneuropathy, neurosyphilis, Ramsay Hunt syndrome	Retrospective review of clinical records
Ahmed et al. (16)	103	Australian	Gentamicin vestibulotoxicity	Gentamicin vestibulotoxicity	Retrospective review of clinical records
Szmulewicz et al. (83)	18	Australian	Characterize and estimate frequency of neuropathy in BV	CABV	Retrospective review of clinical records
Kim et al. (59)	108	Asian	Clinical and laboratory characteristics of BV and propose diagnostic criteria	Idiopathic, ototoxic medication, Menièrés disease, bilateral sequential vestibular neuritis, head trauma, bilateral chronic otitis media, autoimmune disorder, neurologic diseases	Retrospective review of clinical records
Janssen et al. (54)	10	European	Effects of vibrotactile biofeedback	Not specified	Not specified
Zingler et al. (98)	255	European	Etiology, course of manifestations, associated symptoms	Idiopathic, ototoxic medication, Menièrés disease, meningitis/ encephalitis/ cerebellitis, spinocerebellar ataxia/ episodic ataxia/ multiple system atrophy, autoimmune disease, deficit of vitamin B12/ folic acid, Creutzfeldt-Jakob disease, Cogan's syndrome, miscellaneous, positive family history for inner ear diseases,	Retrospective review of clinical records
Zingler et al. (99)	82	European	Frequency and degree of recovery or worsening of vestibular function	Idiopathic, ototoxic medication, meningoencephalitis, autoimmune disorder, Menièrés disease, miscellaneous causes	Standardized detailed history
Zingler et al. (97)	Idem Zingler 2009	European	Determine causative factors and epidemiology	Idem Zingler 2009	Idem Zingler 2009
Ishiyama et al. (50)	35	North-American	Gentamicin ototoxicity	Ototoxic medication (gentamycin)	Questionnaire
Brandt et al. (24)	10	European	MRI and spatial memory and navigation	Neurofibromatosis type 2: bilateral labyrinthectomy	Not specified

(Continued)

Table A1 | Specification of the included clinical studies *(Continued)*

Reference	No. patients with BV	Continent study	Research topic	Etiologies of BV	Method of symptom collection
Schautzer et al. (74)	Idem Brandt 2005	European	Spatial memory	Idem Brandt 2005	Idem Brandt 2005
Wiest et al. (93)	4	North-American	Vestibulo-ocular reflex	Idiopathic	Not specified
Brown et al. (27)	13	North-American	Effect of physical therapy	Ototoxic medication, idiopathic	Retrospective review of clinical records
Baloh et al. (19)	7	North-American	Age-related changes in the vestibulo-ocular reflex	Ototoxic medication, idiopathic	Not specified
Herdman et al.(45)	45	North-American	Falls	Not specified	Interview about falls
Grunfeld et al. (44)	12	European	Design of oscillopsia questionnaire	Idiopathic, bacterial meningitis, neurofibromatosis type 2 with removal of acoustic neuroma, idiopathic cerebellar-vestibular degeneration	Questionnaire
Gill-body et al. (42)	44	North-American	Balance impairment, functional performance and disability	Ototoxic medication, idiopathic, degeneration, autoimmune disease, sequential vestibular neuronitis, otosyphyllis	Patient reported
Gillespie and Minor (43)	35	North-American	Prognosis	Ototoxic medication, idiopathic, neuritis, congenital, ischemic, autoimmune, neurodegenerative, Menièrés disease	Retrospective review of clinical records
Rinne et al. (69)	53	European	Etiologies	Neurological (cerebellar, neuropathy, meningitis), idiopathic, ototoxic medication, autoimmune disorder, otologic (Menièrés disease, temporal bone fracture, Usher's syndrome), neoplastic (lymphoma, leukaemie, hypernephroma)	Retrospective review of clinical records
Lekhel et al. (60)	11	European	Postural responses to vibration of neck muscles	Idiopathic, meningitis and ototoxic medication, ototoxic medication, bilateral neurectomy for neurofibromatosis type 2, Menièrés disease	Not specified
Sargent et al. (73)	13	North-American	Idiopathic BV laboratory tests	Idiopathic	Interview
Lempert et al. (61)	14	European	Contribution of otolith-ocular reflexes to visual stability	Meningitis, ototoxic medication, neurosarcoidosis, neurofibromatosis type 2 with removal of bilateral acoustic neuromas, idiopathic	Patients were asked for presence of oscillopsia
Baloh et al. (22)	22	North-American	Idiopathic BV	Idiopathic	Interview
Honrubia et al. (48)	9	North-American	Vestibulo-ocular reflex	Ototoxic medication, luetic labyrinthitis, unknown inner ear lesion	Not specified
Baloh et al. (20)	5	North-American	Vestibulo-ocular reflex	Ototoxic medication, luetic labyrinthitis, Menièrés disease, Cogan's syndrome, otosclerosis, idiopathic	Not Specified

Table A2 | Specifications of the included case reports

Reference	No. patients with BV	Continent study	Research topic	Etiologies	Method of symptom collection
Wester et al. (92)	4	North-American	Vestibular migraine and BV	Migraine	Medical records
van Leeuwen et al. (89)	1	European	BV and Lyme disease	Lyme borreliosis	Patient reported
Tang et al. (84)	1	North-American	BV and chronic myeloid leukemia	Chronic myeloid leukemia	Patient reported
Rust et al. (72)	1	European	Cerebellar ataxia, neuropathy and vestibular areflexia	Cerebellar ataxia with neuropathy and bilateral vestibular areflexia syndrome (CANVAS)	Patient reported
Ruehl and Guerkov (71)	1	European	Amiodarone and BV	Amiodarone	Patient reported
Jung et al. (55)	3	Asian	Heat exposure and BV	Heat exposure	Patient reported
Finn et al. (37)	1	European	BV with tullio phenomenon	Not specified	Patient reported
Aran Yoo and Kattah (18)	1	North-American	MRI and video head impulse test	Not specified	Patient reported
Yetiser (95)	1	European	Internal auditory canal metastasis	Internal auditory canal metastasis	Patient reported
van Leeuwen et al. (88)	1	European	Sensory ataxic neuropathy, dysarthria and ophthalmoparesis and BV	Genetic defect	Patient reported
Albernaz and Cusin (17)	1	South-American	Video head impulse test	Mannitol	Patient reported
Wong and Abdul Kadir (94)	1	Asian	BV and chronic subjective dizziness	Minor head injury	Patient reported
Kang et al. (57)	1	Asian	Video head impulse test	Superficial siderosis	Patient reported
Castellucci et al. (29)	1	European	BV and Waldenström's macroglobulinemia	Waldenström's macroglobinemia	Patient reported
Wenzel et al. (91)	4	North-American	Vestibular atelectasis	Idiopathic	Patient reported
van Kerckhoven et al. (87)	1	European	Treatment of vertigo with visual illusions	Ototoxic medication	Patient reported
Choi, et al. (31)	3	Asian	Combined peripheral and central BV	Wernicke encephalopathy, CANVAS	Patient reported
Baxter and Agrawal (23)	1	North-American	BV and Turner Syndrome	Turner syndrome	Patient reported
Hirvonen and Aalto (47)	1	European	BV and Cogan's syndrome	Cogan's syndrome	Patient reported
Hertel et al. (46)	2	European	Toxicity of penicillin and aspirin therapy	Ototoxic medication	Patient reported
van de Berg et al. (86)	1	European	Vestibular implant	Meningitis	Patient reported
Spiegel et al. (79)	1	European	Aminopyridine and BV	Aminopyridine	Patient reported
Chen et al. (30)	1	Asian	Interactive wiimote gaze stabilization	Not specified	Patient reported
Smith et al. (78)	1	North-American	BV in neurosacoidosis	Neurosarcoidosis	Patient reported
Kagoya et al. (56)	1	Asian	Cephalic tetanus and BV	Cephalic tetanus	Patient reported
Yukawa et al. (96)	2	Asian	BV and inner ear antibodies	Autoimmune disorder	Patient reported
Robinson et al. (70)	1	North-American	Gentamicin and BV	Ototoxic medication	Patient reported
MacDougall et al. (62)	3	Australian	Driving and BV	Ototoxic medication, cerebellar-pontine angle tumor, traumatic subdural haemorrage	Patient reported
Jahn et al. (51)	1	European	Creutzfeldt-Jakob Disease and BV	Creutzfeldt-Jakob disease	Patient reported
Fujimoto et al. (39)	3	Asian	Idiopathic BV	Idiopathic	Patient reported
Brantberg andLofqvist (25)	5	European	BV and vestibular evoked myogenic potentials	Idiopathic	Patient reported
Tuo et al. (85)	1	Asian	Whiplash-associated disorders and BV	Trauma	Patient reported
Fujimoto et al. (38)	2	Asian	Idiopathic BV	Idiopathic	Patient reported
Agrup et al. (15)	2	European	Autoantibodies against inner ear compartments and BV	Autoimmune disorder	Patient reported
Strupp et al. (80)	1	European	BV and aspirin	Aspirin	Patient reported
Schüler et al. (77)	1	European	Autoimmune BV	Autoimmune disorder	Patient reported
Bringoux et al. (26)	4	European	Perception of pitch and roll in BV	Ototoxic medication, idiopathic, trauma, post-surgical	Patient reported
Pollak et al. (68)	1	European	BV and carcinomatous meningitis	Meningitis	Patient reported
Matsuzaki and Murofushi (64)	3	Asian	Vestibular evoked myogenic potentials in idiopathic BV	Idiopathic	Patient reported
Calder and Jacobson (28)	2	North-American	Acquired BV	Ototoxic medication	Patient reported
Minor (65)	1	North-American	Gentamicin and BV	Ototoxic medication	Patient reported
Acierno et al. (14)	1	North-American	Oscillopsia in idiopathic BV	Idiopathic	Patient reported
Nuti et al. (67)	4	European	BV and vertebrobasilar dolichoectasia	Vertebrobasilar dolichoectasia	Patient reported
Constantinescu et al. (33)	2	European	Vestibular evoked potentials and BV	Jaffé-Lichtenstein Syndrome, trauma	Patient reported
Baloh et al. (21)	3	North-American	Familial BV	Familial	Patient reported
Durrant and Furman (36)	3	North-American	Long-latency rotational evoked potentials and BV	Meningitis, peritoneal dialysis, Klinefelter syndrome	Patient reported
Hughes et al. (49)	4	North-American	Autoimmune BV	Autoimmune disorder	Patient reported

Table A3 | Classification of the terms used in the publications about BV

Classified terms	Terms used in publications
Chronic Dizziness	Dizziness<br>Dizziness during head movements and locomotion<br>Drunkenness<br>Light-headedness<br>Permanent drunkenness<br>Felt drunk and walking into walls
Recurrent Vertigo	Vertigo<br>Episodes of rotatory vertigo<br>Spells of vertigo<br>Spinning spells
Imbalance	Imbalance<br>Persistent unsteadiness<br>Unsteady<br>Unsteadiness<br>Unsteadiness of gait<br>Gait unsteadiness<br>Instability of gait<br>Ataxia<br>Disequilibrium<br>Difficulty in walking<br>Unsteady while walking<br>Not able to stand or walk for long periods of time<br>Impaired balance and gait<br>Off balance<br>Balance disturbances<br>Postural instability<br>Postural unsteadiness<br>Loss of balance<br>Unstable when he was moving
Darkness	Difficulty walking in the dark<br>Worse(ned) in the dark<br>Particularly in the dark<br>Worse at night<br>More important in darkness<br>Aggravation in the dark<br>Especially in darkness<br>Closed his eyes<br>Increase postural imbalance in darkness
Uneven ground	Difficulty walking on uneven surfaces<br>On uneven ground<br>Ground is uneven<br>On a rough terrain<br>On unlevel ground
Oscillopsia	Oscillopsia<br>during locomotion<br>during head and body movements<br>on locomotion<br>Visual blurring<br>on locomotion<br>Blurred vision<br>during head movement or when walking<br>initiated by head movement<br>during head movements<br>Blurred of fuzzy vision<br>Walking-induced vertical oscillopsia<br>Apparent motion of the visual scene<br>Shaking movement of environment<br>Seeing the world bounce and having to stop to read signs<br>Jumping of vision

(Continued)

Classified terms	Terms used in publications
Hearing loss	Hearing loss<br>Hearing loss except for presbyacusis<br>Slight - Profound hearing loss(incl. deafness)<br>Hearing impairment<br>Impaired hearing<br>Noticeable change in hearing<br>Associated hearing loss<br>Disturbances of hearing<br>Hearing problems<br>Audiometric data<br>Deaf
Tinnitus	Tinnitus

REFERENCES

1. Hain TC, Cherchi M, Yacovino DA. Bilateral vestibular loss. Semin Neurol. 2013;33(3):195-203.
2. Lucieer F, Vonk P, Guinand N, Stokroos R, Kingma H, van de Berg R. Bilateral Vestibular Hypofunction: Insights in Etiologies, Clinical Subtypes, and Diagnostics. Front Neurol. 2016;7:26.
3. Strupp M, Kim JS, Murofushi T, Straumann D, Jen JC, Rosengren SM, et al. Bilateral vestibulopathy: Diagnostic criteria Consensus document of the Classification Committee of the Barany Society. J Vestib Res. 2017;27(4):177-89.
4. van de Berg R, van Tilburg M, Kingma H. Bilateral Vestibular Hypofunction: Challenges in Establishing the Diagnosis in Adults. ORL J Otorhinolaryngol Relat Spec. 2015;77(4):197-218.
5. Miffon M, Guyot JP. Difficulties Faced by Patients Suffering from Total Bilateral Vestibular Loss. ORL J Otorhinolaryngol Relat Spec. 2015;77(4):241-7.
6. Guinand N, Boselie F, Guyot JP, Kingma H. Quality of life of patients with bilateral vestibulopathy. Ann Otol Rhinol Laryngol. 2012;121(7):471-7.
7. Hanes DA, McCollum G. Cognitive-vestibular interactions: a review of patient difficulties and possible mechanisms. J Vestib Res. 2006;16(3):75-91.
8. McCall AA, Yates BJ. Compensation following bilateral vestibular damage. Front Neurol. 2011;2:88.
9. Gofrit SG, Mayler Y, Eliashar R, Bdolah-Abram T, Ilan O, Gross M. The Association Between Vestibular Physical Examination, Vertigo Questionnaires, and the Electronystagmography in Patients With Vestibular Symptoms. Ann Otol Rhinol Laryngol. 2017;126(4):315-21.
10. Rothrock NE, Kaiser KA, Cella D. Developing a valid patient-reported outcome measure. Clin Pharmacol Ther. 2011;90(5):737-42.
11. Dawson J, Doll H, Fitzpatrick R, Jenkinson C, Carr AJ. The routine use of patient reported outcome measures in healthcare settings. Brit Med J. 2010;340.
12. Moher D, Liberati A, Tetzlaff J, Altman DG, Group P. Preferred reporting items for systematic reviews and meta-analyses: the PRISMA statement. Int J Surg. 2010;8(5):336-41.
13. McHugh ML. Interrater reliability: the kappa statistic. Biochem Med (Zagreb). 2012;22(3):276-82.
14. Acierno MD, Trobe JD, Shepard NT, Cornblath WT, Disher MJ. Two types of oscillopsia in a patient with idiopathic vestibulopathy. J Neuroophthalmol. 1997;17(2):92-4.
15. Agrup C, Keir G, Thompson EJ, Bronstein AM. Systemic autoantibodies against discrete inner ear compartments in bilateral vestibular loss. Neurology. 2005;65(1):167.
16. Ahmed RM, Hannigan IP, MacDougall HG, Chan RC, Halmagyi GM. Gentamicin ototoxicity: a 23-year selected case series of 103 patients. Med J Aust. 2012;196(11):701-4.
17. Albernaz PL, Cusin FS. The Video Head Impulse Test in a Case of Suspected Bilateral Loss of Vestibular Function. Int Arch Otorhinolaryngol. 2016;20(1):84-6.
18. Aran Yoo BS, Kattah JC. Superficial siderosis syndrome with progressive hearing loss and bilateral vestibular failure, 51 years after a neurosurgical procedure: diagnostic value of combined MRI and video head impulse test. J Neurol. 2017;264(2):391-3.
19. Baloh RW, Enrietto J, Jacobson KM, Lin A. Age-related changes in vestibular function: a longitudinal study. Ann N Y Acad Sci. 2001;942:210-9.
20. Baloh RW, Honrubia V, Yee RD, Hess K. Changes in the human vestibulo-ocular reflex after loss of peripheral sensitivity. Ann Neurol. 1984;16(2):222-8.
21. Baloh RW, Jacobson K, Fife T. Familial vestibulopathy: a new dominantly inherited syndrome. Neurology. 1994;44(1):20-5.
22. Baloh RW, Jacobson K, Honrubia V. Idiopathic bilateral vestibulopathy. Neurology. 1989;39(2 Pt 1):272-5.
23. Baxter M, Agrawal Y. Vestibular dysfunction in Turner syndrome: a case report. Otol Neurotol. 2014;35(2):294-6.
24. Brandt T, Schautzer F, Hamilton DA, Bruning R, Markowitsch HJ, Kalla R, et al. Vestibular loss causes hippocampal atrophy and impaired spatial memory in humans. Brain. 2005;128(Pt 11):2732-41.
25. Brantberg K, Lofqvist L. Preserved vestibular evoked myogenic potentials (VEMP) in some patients with walking-induced oscillopsia due to bilateral vestibulopathy. J Vestib Res. 2007;17(1):33-8.
26. Bringoux L, Schmerber S, Nougier V, Dumas G, Barraud PA, Raphel C. Perception of slow pitch and roll body tilts in bilateral labyrinthine-defective subjects. Neuropsychologia. 2002;40(4):367-72.
27. Brown KE, Whitney SL, Wrisley DM, Furman JM. Physical therapy outcomes for persons with bilateral vestibular loss. Laryngoscope. 2001;111(10):1812-7.
28. Calder JH, Jacobson GP. Acquired bilateral peripheral vestibular system impairment: rehabilitative options and potential outcomes. J Am Acad Audiol. 2000;11(9):514-21.
29. Castellucci A, Piras G, Brandolini C, Modugno GC, Ferri GG. Waldenstrom's macroglobulinemia presenting with bilateral vestibular loss: a case report. Braz J Otorhinolaryngol. 2015;81(5):571-5.
30. Chen PY, Hsieh WL, Wei SH, Kao CL. Interactive wiimote gaze stabilization exercise training system for patients with vestibular hypofunction. J Neuroeng Rehabil. 2012;9:77.
31. Choi SY, Kee HJ, Park JH, Kim HJ, Kim JS. Combined peripheral and central vestibulopathy. J Vestib Res. 2014;24(5-6):443-51.
32. Choi SY, Kim HJ, Kim JS. Chasing dizzy chimera: Diagnosis of combined peripheral and central vestibulopathy. J Neurol Sci. 2016;371:69-78.
33. Constantinescu L, Schneider D, Claussen C. Vestibular Evoked Potentials in Two Patients with Bilateral Vestibular Loss. Int Tinnitus J. 1996;2:45-57.
34. de Waele C, Shen Q, Magnani C, Curthoys IS. A Novel Saccadic Strategy Revealed by Suppression Head Impulse Testing of Patients with Bilateral Vestibular Loss. Front Neurol. 2017;8:419.
35. Deroualle D, Toupet M, van Nechel C, Duquesne U, Hautefort C, Lopez C. Anchoring the Self to the Body in Bilateral Vestibular Failure. PLoS One. 2017;12(1):e0170488.
36. Durrant JD, Furman JM. Long-latency rotational evoked potentials in subjects with and without bilateral vestibular loss. Electroencephalogr Clin Neurophysiol. 1988;71(4):251-6.
37. Finn S, Dietzek M, Karvouniari P, Klingner CM, Neumann R, Guntinas-Lichius O, et al. Bilateral vestibulopathy with positive Tullio phenomenon. Laryngoscope. 2017.
38. Fujimoto C, Iwasaki S, Matsuzaki M, Murofushi T. Lesion site in idiopathic bilateral vestibulopathy: a galvanic vestibular-evoked myogenic potential study. Acta Otolaryngol. 2005;125(4):430-2.
39. Fujimoto C, Murofushi T, Chihara Y, Suzuki M, Yamasoba T, Iwasaki S. Novel subtype of idiopathic bilateral vestibulopathy: bilateral absence of vestibular evoked myogenic potentials in the presence of normal caloric responses. J Neurol. 2009;256(9):1488-92.
40. Fujimoto C, Murofushi T, Sugasawa K, Chihara Y, Ushio M, Yamasoba T, et al. Bilateral vestibulopathy with dissociated deficits in the superior and inferior vestibular systems. Ann Otol Rhinol Laryngol. 2012;121(6):383-8.
41. Ghulyan-Bedikian V, Paolino M, Paolino F. Short-term retention effect of rehabilitation using head position-based electrotactile feedback to the tongue: influence of vestibular loss and old-age. Gait Posture. 2013;38(4):777-83.
42. Gill-Body KM, Beninato M, Krebs DE. Relationship among balance impairments, functional performance, and disability in people with peripheral vestibular hypofunction. Phys Ther. 2000;80(8):748-58.
43. Gillespie MB, Minor LB. Prognosis in bilateral vestibular hypofunction. Laryngoscope. 1999;109(1):35-41.
44. Grunfeld EA, Morland AB, Bronstein AM, Gresty MA. Adaptation to oscillopsia: a psychophysical and questionnaire investigation. Brain. 2000;123 (Pt 2):277-90.
45. Herdman SJ, Blatt P, Schubert MC, Tusa RJ. Falls in patients with vestibular deficits. Am J Otol. 2000;21(6):847-51.
46. Hertel S, Schwaninger M, Helmchen C. Combined toxicity of penicillin and aspirin therapy may elicit bilateral vestibulopathy. Clin Neurol Neurosurg. 2013;115(7):1114-6.
47. Hirvonen TP, Aalto H. Recovery of bilateral vestibular loss in Cogan's syndrome--a case report. Otol Neurotol. 2013;34(9):1736-8.
48. Honrubia V, Marco J, Andrews J, Minser K, Yee RD, Baloh RW. Vestibulo-ocular reflexes in peripheral labyrinthine lesions: III. Bilateral dysfunction. Am J Otolaryngol. 1985;6(5):342-52.
49. Hughes GB, Kinney SE, Hamid MA, Barna BP, Calabrese LH. Autoimmune vestibular dysfunction: preliminary report. Laryngoscope. 1985;95(8):893-7.
50. Ishiyama G, Ishiyama A, Kerber K, Baloh RW. Gentamicin ototoxicity: clinical features and the effect on the human vestibulo-ocular reflex. Acta Otolaryngol. 2006;126(10):1057-61.
51. Jahn K, Arbusow V, Zingler VC, Strupp M, Kretzschmar HA, Brandt T. Bilateral vestibular failure as an early sign in Creutzfeldt-Jakob disease. Ann N Y Acad Sci. 2009;1164:390-3.
52. Jandl NM, Sprenger A, Wojak JF, Gottlich M, Munte TF, Kramer UM, et al. Dissociable cerebellar activity during spatial navigation and visual memory in bilateral vestibular failure. Neuroscience. 2015;305:257-67.
53. Jansen NL, Feuerecker R, Becker-Bense S, Zwergal A, Wulff M, Xiong G, et al. Assessment of cerebral dopamine D 2/3 formula-receptors in patients with bilateral vestibular failure. J Vestib Res. 2014;24(5-6):403-13.
54. Janssen M, Stokroos R, Aarts J, van Lummel R, Kingma H. Salient and placebo vibrotactile feedback are equally effective in reducing sway in bilateral vestibular loss patients. Gait Posture. 2010;31(2):213-7.
55. Jung I, Choi SY, Kim HJ, Kim JS. Delayed vestibulopathy after heat exposure. J Neurol. 2017;264(1):49-53.
56. Kagoya R, Iwasaki S, Chihara Y, Ushio M, Tsuji S, Murofushi T, et al. Cephalic tetanus presenting as acute vertigo with bilateral vestibulopathy. Acta Otolaryngol. 2011;131(3):334-6.
57. Kang KW, Lee C, Kim SH, Cho HH, Lee SH. Bilateral Vestibulopathy Documented by Video Head Impulse Tests in Superficial Siderosis. Otol Neurotol. 2015;36(10):1683-6.
58. Kapoula Z, Gaertner C, Yang Q, Denise P, Toupet M. Vergence and Standing Balance in Subjects with Idiopathic Bilateral Loss of Vestibular Function. PLoS One. 2013;8(6):e66652.
59. Kim S, Oh YM, Koo JW, Kim JS. Bilateral vestibulopathy: clinical characteristics and diagnostic criteria. Otol Neurotol. 2011;32(5):812-7.

60. Lekhel H, Popov K, Bronstein A, Gresty M. Postural responses to vibration of neck muscles in patients with uni- and bilateral vestibular loss. Gait Posture. 1998;7(3):228-36.
61. Lempert T, Gianna CC, Gresty MA, Bronstein AM. Effect of otolith dysfunction. Impairment of visual acuity during linear head motion in labyrinthine defective subjects. Brain. 1997;120 (Pt 6):1005-13.
62. MacDougall HG, Moore ST, Black RA, Jolly N, Curthoys IS. On-road assessment of driving performance in bilateral vestibular-deficient patients. Ann N Y Acad Sci. 2009;1164:413-8.
63. Martin T, Moussay S, Bulla I, Bulla J, Toupet M, Etard O, et al. Exploration of Circadian Rhythms in Patients with Bilateral Vestibular Loss. PLoS One. 2016;11(6):e0155067.
64. Matsuzaki M, Murofushi T. Vestibular evoked myogenic potentials in patients with idiopathic bilateral vestibulopathy. Report of three cases. ORL J Otorhinolaryngol Relat Spec. 2001;63(6):349-52.
65. Minor LB. Gentamicin-induced bilateral vestibular hypofunction. JAMA. 1998;279(7):541-4.
66. Moon M, Chang SO, Kim MB. Diverse clinical and laboratory manifestations of bilateral vestibulopathy. Laryngoscope. 2017;127(1):E42-E9.
67. Nuti D, Passero S, Di Girolamo S. Bilateral vestibular loss in vertebrobasilar dolichoectasia. J Vestib Res. 1996;6(2):85-91.
68. Pollak L, Milo R, Kossych V, Rabey MJ, Shapira E. Bilateral vestibular failure as a unique presenting sign in carcinomatous meningitis: case report. J Neurol Neurosurg Psychiatry. 2001;70(5):704-5.
69. Rinne T, Bronstein AM, Rudge P, Gresty MA, Luxon LM. Bilateral loss of vestibular function: clinical findings in 53 patients. J Neurol. 1998;245(6-7):314-21.
70. Robinson BS, Cook JL, Richburg CM, Price SE. Use of an electrotactile vestibular substitution system to facilitate balance and gait of an individual with gentamicin-induced bilateral vestibular hypofunction and bilateral transtibial amputation. J Neurol Phys Ther. 2009;33(3):150-9.
71. Ruehl RM, Guerkov R. Amiodarone-induced gait unsteadiness is revealed to be bilateral vestibulopathy. Eur J Neurol. 2017;24(2):e7-e8.
72. Rust H, Peters N, Allum JHJ, Wagner B, Honegger F, Baumann T. VEMPs in a patient with cerebellar ataxia, neuropathy and vestibular areflexia (CANVAS). J Neurol Sci. 2017;378:9-11.
73. Sargent EW, Goebel JA, Hanson JM, Beck DL. Idiopathic bilateral vestibular loss. Otolaryngol Head Neck Surg. 1997;116(2):157-62.
74. Schautzer F, Hamilton D, Kalla R, Strupp M, Brandt T. Spatial memory deficits in patients with chronic bilateral vestibular failure. Ann N Y Acad Sci. 2003;1004:316-24.
75. Schlick C, Schniepp R, Loidl V, Wuehr M, Hesselbarth K, Jahn K. Falls and fear of falling in vertigo and balance disorders: A controlled cross-sectional study. J Vestib Res. 2016;25(5-6):241-51.
76. Schniepp R, Schlick C, Schenkel F, Pradhan C, Jahn K, Brandt T, et al. Clinical and neurophysiological risk factors for falls in patients with bilateral vestibulopathy. J Neurol. 2017;264(2):277-83.
77. Schuler O, Strupp M, Arbusow V, Brandt T. A case of possible autoimmune bilateral vestibulopathy treated with steroids. J Neurol Neurosurg Psychiatry. 2003;74(6):825.
78. Smith JH, Stovall KC, Coons S, Fife TD. Bilateral vestibular hypofunction in neurosarcoidosis: a case report. Ear Nose Throat J. 2011;90(1):E1-3.
79. Spiegel R, Kalla R, Classen J, Bardins S, Anciaes da Silva F, Farahmand P, et al. Aminopyridine treatment in a patient with bilateral vestibular failure and cryptogenic downbeat nystagmus. J Neuroophthalmol. 2012;32(2):190.
80. Strupp M, Jahn K, Brandt T. Another adverse effect of aspirin: bilateral vestibulopathy. J Neurol Neurosurg Psychiatry. 2003;74(5):691.
81. Suarez H, Sotta G, San Roman C, Arocena S, Ferreira E, Geisinger D, et al. Postural response characterization in elderly patients with bilateral vestibular hypofunction. Acta Otolaryngol. 2013;133(4):361-7.
82. Swanenburg J, Zurbrugg A, Straumann D, Hegemann SCA, Palla A, de Bruin ED. A pilot study investigating the association between chronic bilateral vestibulopathy and components of a clinical functional assessment tool. Physiother Theory Pract. 2017;33(6):454-61.
83. Szmulewicz DJ, Waterston JA, Halmagyi GM, Mossman S, Chancellor AM, McLean CA, et al. Sensory neuropathy as part of the cerebellar ataxia neuropathy vestibular areflexia syndrome. Neurology. 2011;76(22):1903-10.
84. Tang L, Schubert M, Marlowe A, Weinreich H. Bilateral Hearing and Vestibular Loss in a Patient With Untreated Chronic Myeloid Leukemia. JAMA Otolaryngol Head Neck Surg. 2017;143(7):736-7.
85. Tuo KS, Cheng YY, Kao CL. Vestibular rehabilitation in a patient with whiplash-associated disorders. J Chin Med Assoc. 2006;69(12):591-5.
86. van de Berg R, Guinand N, Guyot JP, Kingma H, Stokroos RJ. The modified ampullar approach for vestibular implant surgery: feasibility and its first application in a human with a long-term vestibular loss. Front Neurol. 2012;3:18.
87. van Kerckhoven G, Mert A, De Ru JA. Treatment of vertigo and postural instability using visual illusions. J Laryngol Otol. 2014;128(11):1005-7.
88. van Leeuwen RB, Smits BW, Rodenburg RJ, van Engelen BG. Bilateral Vestibulopathy Aggravates Balance and Gait Disturbances in Sensory Ataxic Neuropathy, Dysarthria, and Ophthalmoparesis: A Case Report. J Clin Neuromuscul Dis. 2016;18(1):34-6.
89. van Leeuwen RB, van Kooten B, de Cock AF. Bilateral vestibular hypofunction and Lyme disease: a causal link? Acta Neurol Belg. 2017;117(1):367-8.
90. Ward BK, Agrawal Y, Hoffman HJ, Carey JP, Della Santina CC. Prevalence and impact of bilateral vestibular hypofunction: results from the 2008 US National Health Interview Survey. JAMA Otolaryngol Head Neck Surg. 2013;139(8):803-10.
91. Wenzel A, Ward BK, Schubert MC, Kheradmand A, Zee DS, Mantokoudis G, et al. Patients with vestibular loss, tullio phenomenon, and pressure-induced nystagmus: vestibular atelectasis? Otol Neurotol. 2014;35(5):866-72.
92. Wester JL, Ishiyama A, Ishiyama G. Recurrent Vestibular Migraine Vertigo Attacks Associated With the Development of Profound Bilateral Vestibulopathy: A Case Series. Otol Neurotol. 2017;38(8):1145-8.
93. Wiest G, Demer JL, Tian J, Crane BT, Baloh RW. Vestibular function in severe bilateral vestibulopathy. J Neurol Neurosurg Psychiatry. 2001;71(1):53-7.
94. Wong RS, Abdul Kadir SY. An unusual case of bilateral vestibulopathy, chronic subjective dizziness and spondyloarthropathy. Gen Hosp Psychiatry. 2015;37(4):372 e3-4.
95. Yetiser S. Bilateral Cochleovestibulopathy Due to Internal Auditory Canal Metastasis in a Patient with Stomach Cancer. J Int Adv Otol. 2016;12(3):353-5.
96. Yukawa K, Hagiwara A, Ogawa Y, Nishiyama N, Shimizu S, Kawaguchi S, et al. Bilateral progressive hearing loss and vestibular dysfunction with inner ear antibodies. Auris Nasus Larynx. 2010;37(2):223-8.
97. Zingler VC, Cnyrim C, Jahn K, Weintz E, Fernbacher J, Frenzel C, et al. Causative factors and epidemiology of bilateral vestibulopathy in 255 patients. Ann Neurol. 2007;61(6):524-32.
98. Zingler VC, Weintz E, Jahn K, Huppert D, Cnyrim C, Brandt T, et al. Causative factors, epidemiology, and follow-up of bilateral vestibulopathy. Ann N Y Acad Sci. 2009;1164:505-8.
99. Zingler VC, Weintz E, Jahn K, Mike A, Huppert D, Rettinger N, et al. Follow-up of vestibular function in bilateral vestibulopathy. J Neurol Neurosurg Psychiatry. 2008;79(3):284-8.
100. Rybak LP, Whitworth CA. Ototoxicity: therapeutic opportunities. Drug Discov Today. 2005;10(19):1313-21.
101. Cruickshanks KJ, Tweed TS, Wiley TL, Klein BE, Klein R, Chappell R, et al. The 5-year incidence and progression of hearing loss: the epidemiology of hearing loss study. Arch Otolaryngol Head Neck Surg. 2003;129(10):1041-6.
102. Balaban CD. Projections from the parabrachial nucleus to the vestibular nuclei: potential substrates for autonomic and limbic influences on vestibular responses. Brain Res. 2004;996(1):126-37.
103. Highstein SM, Holstein GR. The anatomical and physiological framework for vestibular prostheses. Anat Rec (Hoboken). 2012;295(11):2000-9.
104. Holstein GR, Friedrich VL, Jr., Kang T, Kukielka E, Martinelli GP. Direct projections from the caudal vestibular nuclei to the ventrolateral medulla in the rat. Neuroscience. 2011;175:104-17.
105. Grill E, Strupp M, Muller M, Jahn K. Health services utilization of patients with vertigo in primary care: a retrospective cohort study. J Neurol. 2014;261(8):1492-8.
106. Sun DQ, Ward BK, Semenov YR, Carey JP, Della Santina CC. Bilateral Vestibular Deficiency: Quality of Life and Economic Implications. JAMA Otolaryngol Head Neck Surg. 2014;140(6):527-34.
107. Cutfield NJ, Scott G, Waldman AD, Sharp DJ, Bronstein AM. Visual and proprioceptive interaction in patients with bilateral vestibular loss. Neuroimage Clin. 2014;4:274-82.
108. Guerraz M, Yardley L, Bertholon P, Pollak L, Rudge P, Gresty MA, et al. Visual vertigo: symptom assessment, spatial orientation and postural control. Brain. 2001;124(Pt 8):1646-56.
109. Jacobson GP, Newman CW. The development of the Dizziness Handicap Inventory. Arch Otolaryngol Head Neck Surg. 1990;116(4):424-7.
110. Cohen HS, Kimball KT. Development of the vestibular disorders activities of daily living scale. Arch Otolaryngol Head Neck Surg. 2000;126(7):881-7.
111. Newman-Toker DE, Edlow JA. TiTrATE: A Novel, Evidence-Based Approach to Diagnosing Acute Dizziness and Vertigo. Neurol Clin. 2015;33(3):577-99, viii.
